# CXCR4^+^ cells are increased in lung tissue of patients with idiopathic pulmonary fibrosis

**DOI:** 10.1186/s12931-020-01467-0

**Published:** 2020-08-26

**Authors:** Jade Jaffar, Kate Griffiths, Sara Oveissi, Mubing Duan, Michael Foley, Ian Glaspole, Karen Symons, Louise Organ, Glen Westall

**Affiliations:** 1 Dept. of Allergy, Immunology and Respiratory Medicine, The Alfred Hospital, Melbourne, Australia; 2 Dept. of Immunology and Pathology, Monash University, Melbourne, Australia; 3 N.H.M.R.C. Centre of Research Excellence in Pulmonary Fibrosis, Sydney, Australia; 4 Walter and Eliza Hall Institute of Medical Research, Parkville, Melbourne, Australia; 5 The Department of Biochemistry and Genetics, La Trobe Institute for Molecular Science, La Trobe University, Bundoora, Melbourne, Australia; 6 AdAlta Limited, Bundoora, Australia; 7 Dept. of Medicine, Monash University, Melbourne, Australia

**Keywords:** CXCR4, CXCL12, Pulmonary fibrosis, Interstitial lung disease

## Abstract

**Background:**

CXCR4, a transmembrane-receptor located on epithelial cells that is activated by CXCL12, may have a role in IPF via migration of CXCR4^+^ fibrocytes to the lung. However, its expression has not been fully characterised in idiopathic pulmonary fibrosis (IPF) or other fibrotic interstitial lung diseases (ILDs). CXCL12 is constitutively expressed in the bone marrow, and levels of CXCR4 regulate control of this signalling pathway. The aim of this study was to profile the expression of CXCR4 in lung tissue and peripheral circulation of patients with IPF and other fibrotic ILDs.

**Methods:**

Expression of CXCR4 on peripheral blood mononuclear cells (PBMCs) was examined by flow cytometry in 20 patients with IPF and 10 age-matched non-disease control (NDC) donors. Levels of CXCL12 in human plasma were measured by ELISA. Expression of CXCR4, CXCL12, CD45, and e-cadherin was assessed in IPF (*n* = 10), other fibrotic ILD (*n* = 8) and NDC (n = 10) lung tissue by multiplex immunohistochemistry (OPAL) and slides were scanned using a Vectra 3 scanner. Cells were quantified with computer automated histological analysis software (HALO).

**Results:**

In blood, the number of CXCR4^+^ cells was lower but the level of CXCL12 was higher in patients with IPF compared to NDC donors. Elevated CXCR4 expression was detected in lung tissue from patients with IPF and other fibrotic ILDs compared to NDC. There were higher levels of CXCR4^+^/e-cadherin^+^/CXCL12^+^ (epithelial) cells in IPF lung tissue compared to NDC, but there was no difference in the numbers of CXCR4^+^/CD45^+^/CXCL12^+^ (myeloid) cells between the two groups.

**Conclusions:**

This report demonstrates that CXCR4 is overexpressed not only in IPF but also in other ILDs and expression is particularly prominent within both honeycomb cysts and distal airway epithelium. This observation supports the hypothesis that CXCR4 may drive tissue fibrosis through binding its specific ligand CXCL12. Although CXCR4 expressing cells could be either of epithelial or myeloid origin it appears that the former is more prominent in IPF lung tissue. Further characterization of the cells of the honeycomb cyst may lead to a better understanding of the fibrogenic processes in IPF and other end-stage fibrotic ILDs.

## Background

Idiopathic pulmonary fibrosis (IPF) is a chronic progressive interstitial lung disease (ILD) with few effective drug therapies [1, 2]. An early event in IPF is recurrent epithelial injury resulting in activation of extracellular matrix (ECM)-producing fibroblasts [3]. CXCR4 is a transmembrane protein receptor that is constitutively expressed on epithelial and myeloid cells and is involved in stem cell migration [4]. CXCR4 is activated by the chemokine CXCL12, also known as stromal derived factor (SDF)-1 and in the bone marrow, CXCL12 is constitutively produced in order to maintain hemopoietic stem cells in the bone marrow environment [5].

In the lung, CXCL12 is upregulated upon epithelial injury [6]. Other CXCR4 expressing cells include fibrocytes which are bone marrow-derived, circulating progenitor cells that express the marker CD45, traffic to the lung via CXCR4/CXCL12 signalling and have been implicated in fibrotic ILD [7]. However, an analysis of the specific cell types expressing CXCR4 in IPF has not been attempted. As a result, there is still some uncertainty as to the origin of CXCR4 expressing cells in the diseased lung. In particular, whether CXCR4^+^ cells are derived from lung resident cells perhaps of epithelial origin or do they originate from the peripheral circulation remains elusive.

In a previous study, the i-body CXCR4 antagonist, AD-114, reduced experimental lung fibrosis and decreased IPF lung fibroblast invasion *in-vitro* [8], which supports the targeting of CXCR4 as a therapeutic option in IPF. CXCR4 is best known for its role in diverse disorders such as HIV-1 infection [9], tumour development [10], and acute myeloid leukemia [11]. In the lung, CXCR4 expression is thought to contribute to the cellular trafficking from the vessels into the interstitium and contribute to a number of fibrotic disorders including asthma and pulmonary fibrosis [12]. However, it is not known if circulating cells expressing only CXCR4 could be potential biomarkers of disease progression in IPF. Therefore, the first aim of this study was to investigate whether numbers of CXCR4^+^ cells in the circulation of patients with IPF were elevated compared to age-matched normal controls and if there was any correlation with clinical parameters of disease.

Radiologically, honeycomb lung is defined by clusters of sub-pleural basal cystic air spaces seen on high-resolution computed tomography (HRCT) scans in patients with end-stage fibrotic ILD, and in particular those with the usual interstitial pneumonia (UIP) pattern of fibrosis. Patients with ILDs that share a radiologic and histological pattern of UIP do worse than those that have other patterns such as non-specific interstitial pneumonia (NSIP) [13]. Histologically, honeycomb cysts are lined with cuboidal or ciliated cells that express a variety of epithelial markers such as e-cadherin and basal cell-specific keratins, supporting the hypothesis that honeycomb cysts are derived from the distal airway [14] but further investigation into the cells of the honeycomb cyst is necessary before anti-CXCR4 therapies in IPF can be fully realised.

We have previously published that CXCR4 expression is increased in IPF lung tissue, particularly in airway and honeycomb-associated epithelial cells [8]. However, it is not known whether these cells also express CXCL12, which would imply that an autocrine CXCR4-CXCL12 pathway could be continuously activated in IPF. Therefore, we then aimed to investigate the CXCR4/CXCL12 expression in the lung tissue of patients with IPF, non-IPF fibrotic ILD and NDC.

## Methods

### Patients/healthy donor/s

Patient demographics are described in Table 1 and Table 2.

**Table 1:** Patient demographics of patients with idiopathic pulmonary fibrosis (IPF) in the blood study

	IPF (*n* = 20)
**Median Age, years (SD)**	67 (11)
**Smoking History, n (%)**	14 (70%)
**Median Forced Vital Capacity (FVC), % predicted (SD)**	68 (16)
**Median Transfer Factor of Carbon Monoxide (TLCO), % predicted (SD) NDC**	43 (14)

**Table 2:** Patient demographics of the donors in the tissue study

	(*n* = 10)	IPF (*n* = 10)	ILD (*n* = 8)
**Median Age, years (SD)**	60 (16)	61 (4)	60 (6)
**Smoking History, n (%)**	4 (40%)	7 (70%)	4 (50%)
**Median Forced Vital Capacity (FVC), % predicted (SD)**	n/a	43 (16)	48 (27)
**Median Transfer Factor of Carbon Monoxide (TLCO),** **% predicted (SD**	n/a	17 (8)	43 (13)

NDC non-diseased control, IPF idiopathic pulmonary fibrosis, ILD interstitial lung disease other than IPF. Non-IPF ILD diagnoses included non-specific interstitial pneumonia (NSIP, n = 2), chronic hypersenstitivity pneumonitis (HP, n = 4), connective tissue disease associated ILD (CTD-ILD, *n* = 2)

All patients had been diagnosed at the Alfred Hospital’s lung fibrosis multi-disciplinary team (MDT) meeting according to recommended guidelines [15].

### Blood study population

A total of 20 patients with IPF and 10 NDC volunteers (aged > 50 years) were included. Of the IPF patients, 13 were on antifibrotic medication (Pirfenidone, *n* = 8; Nintedanib, *n* = 5). Age-matched NDC donors were recruited from the Australian Red Cross Blood Donation Centre. Plasma samples from IPF and NDC donors was prepared by fractionation of whole blood.

### Tissue study population

Explant lung tissue was obtained from patients with IPF (*n* = 10 patients) and other interstitial lung diseases (ILDs)(*n* = 8) at the time of lung transplantation. All patients had pathologist-verified histopathology reports of Usual Interstitial Pneumonia (UIP). None of the IPF patients undergoing lung transplantation were on antifibrotic medication. Non-IPF ILD diagnoses included non-specific interstitial pneumonia (NSIP, *n* = 2), chronic hypersensitivity pneumonitis (HP, *n* = 4), connective tissue disease associated ILD (CTD-ILD, n = 2). Tissue from non-disease controls (NDC) were obtained from deceased organ donors whose lungs had been declined for transplantation.

Because IPF typically manifests first in the lung bases and the fibrosis progresses upwards towards the lung apices, we examined tissue from multiple regions of the lungs to take in account this inherent heterogeneity. All lung tissue was obtained from the Alfred Lung Fibrosis Biobank [supported by the National Health and Medical Research Council (NHMRC) Centre for Research Excellence in Pulmonary Fibrosis].

### Flow cytometric analysis

Polymorphonuclear cells were fractioned from whole blood of patients with IPF and NDC donors and frozen for later use. For cell characterization, cells were stained with an antibody cocktail containing antibodies specific for human CXCR4 (R&D systems, USA), CD4 (eBioscience, USA), CD8 (Pharmingen, USA), B-cell marker CD19 (eBioscience, USA), early myeloid marker CD33 (BD Biosciences, USA) and LIVE/DEAD™ Fixable Aqua Dead Cell Stain (ThermoFisher, USA) and analysed by flow cytometry. Only samples containing more than 5000 live cells were included for analysis.

### Enzyme-linked Immunosorbent assay (ELISA)

CXCL12 in plasma was measured by ELISA according to manufacturer’s instructions (IBT Systems, Germany). Briefly, plasma samples were recovered from liquid nitrogen, thawed at room temperature and used undiluted in the assay. Antibody working solutions and CXCL12 standards were prepared on the day of the experiment in kit assay buffer. Test solutions (plasma, CXCL12 standard, or assay buffer only, 50 μl), in duplicate, were added to 100 μl of capture antibody (biotinylated anti-HuSDF1α antibody) + detection antibody (anti-HuSDF1α monoclonal antibody) and incubated for 1 h at RT. Following washing, 100 μl of conjugate working solution (goat anti-mouse IgG peroxidase conjugate) was added to each well. The plate was incubated for a further 1 h at room temperature, washed then developed using TMB-ELISA Substrate Solution (100 μl/well; supplied) for 30 min. The reaction was stopped with 2 M H_2_SO_4_ (50 μl/well; supplied) and the plate was read using a SpectraMax plate reader at 450 nm.

### Immunohistochemistry (IHC)

From formalin-fixed paraffin-embedded tissue blocks, 4 μm thick paraffin sections were immunohistochemically stained for CXCR4 (dilution 1:500, clone UMB2, Cat# ab124824, Abcam, UK), CXCL12 (dilution, 1:500, clone EPR1216, Cat# ab155090, Abcam, UK), E-Cadherin (epithelial marker, dilution 1:1000, clone EP700Y, Cat# ab40772, Abcam, UK) and CD45 (myeloid marker, dilution 1:1000, clone MEM-28, Cat# ab8216, Abcam, UK). Antigen retrieval was performed in pH 6 citrate buffer (Sigma, Australia). After incubation with DakoEnVision secondary reagents (Dako, Australia), positive staining was visualized for brightfield using diaminobenzidine (Dako, Australia). Sections were counterstained in Mayer’s hemotoxylin (Sigma, Australia). All sections for brightfield analysis were stained at the same time. Sections were scanned using an Aperio Scanscope AT Turbo (Leica Biosystems, Australia) and images were captured at a resolution of 0.25 μm/pixel. The extent of overall CXCR4 expression was semi-quantified by rating the intensity and presence of CXCR4 staining across the entire section on a scale of 0 (absent), 1 (low), or 2 (medium – high) by a researcher blinded to the diagnosis of the section.

All sections for multiplex staining in both panels were stained at the same time. For multiplex staining, tyramide signal amplification was performed using Perkin-Elmer’s OPAL dye reagents (diluted 1:100 and incubated for 6 min) in 3 spectrally distinct fluorophores that have non-overlapping fluorescent spectra, allowing the individual antigens to be separated from a single image (Additional Figure 1).

Whole sections were scanned at 4× magnification using the Vectra 3 Quantitative Pathology Imaging System and the entire section was encircled to define the area to be analysed. Regions of interest (ROIs) were then automatically generated that encapsulated the area of the tissue section for subsequent analysis at 20× magnification (with a resolution 0.5 μm) using Phenochart (v 1.0, Perkin Elmer) software (Additional Figure 2). Exposure settings and emission/excitation wavelengths for each of the channels and OPAL dyes are listed in Additional Figure 2.

Sections were sequentially stained with primary antibodies in two panels and cell nuclei was stained with Hoechst in both. Panel 1 consisted of CXCR4, E-Cadherin and CXCL12. Panel 2 consisted of CXCR4, CD45 and CXCL12. The sequence of targets and dye in Panel 1 was CXCR4 (OPAL 520), e-cadherin (OPAL 570), and CXCL12 (OPAL 690). The sequence of targets and dye in Panel 2 was CXCR4 (OPAL 520), CD45 (OPAL 570) and CXCL12 (OPAL 690). Hoechst dye was applied as the last step in both panels.

Multiplex analysis was performed on tissue sections from 7 NDC donors and 7 patients with IPF using HALO imaging processing software (Indica Labs Highplex FL version 3.0) on a total of 2867 images (ROIs) (Table 3). There were 1458 ROIs captured in the e-cadherin panel and 1409 ROIs in the CD45 panel. Additional File 3 details the thresholds used for positive cell quantification. Cells in lung tissue were identified using Hoechst staining and thresholds for positivity of the panel markers were manually set by examining 2 randomly chosen ROIs from a NDC donor and IPF patient (total of 4 images). Additional File 3 shows two ROIs quantified for Panel 1 and Additional File 4 shows two ROIs quantified for Panel 2. Thresholds and corresponding cell phenotype combinations are then applied to all ROIs used in the analysis. Quantification is performed on all the ROIs from each tissue section and data is presented as percentage of Hoechst+ cells counted.

**Table 3:** Summary of quality control parameters for multiplex immunohistochemsitry analysis

E-Cadherin Panel	Normal (*N* = 7)		IPF (*N* = 7)		Mann-Whitney U (Nonparametric)	Effect Size
Variable	Mean1	SD	Mean2	SD	*p*-value	
Number of ROIs in analysis	116	31.5	92	37.9	0.259	
Total number cell-sized objects	193,341	43,830	156,428	59,257	0.209	
Hoechst 33258 Positive Cells	58,854	28,534	48,439	34,558	0.209	
Avg Cell Area (μm^2^)	126.4	8.0	125.1	4.8	0.535	
Avg Cytoplasm Area (μm^2^)	73.0	11.5	75.2	7.6	0.902	
Avg Nucleus Area (μm^2^)	53.4	5.0	49.9	5.1	0.259	
Avg Nucleus Perimeter (μm)	34.4	2.1	33.7	1.8	0.456	
Avg Nucleus Roundness	0.7	0.005	0.7	0.007	0.001**	
Area Analyzed (μm^2^)	5E+ 07	2E+ 07	4E+ 07	2E+ 07	0.383	−2.073
CD45 Panel						
	Normal (*N* = 7)		IPF (*N* = 7)		Mann-Whitney U (Nonparametric)	Effect Size
Variable	Mean1	SD	Mean2	SD	*p*-value	
Number of ROIs in analysis	111	32.4	91	33.5	0.318	
Total number cell-sized objects	186,596	52,479	153,307	53,860	0.318	
Hoechst 33258 Positive Cells	52,770	31,528	41,252	31,069	0.456	
Avg Cell Area (μm^2^)	125.0	6.9	123.6	5.9	0.902	
Avg Cytoplasm Area (μm^2^)	71.9	10.4	73.0	8.8	1.000	
Avg Nucleus Area (μm^2^)	53.0	4.8	50.6	4.3	0.383	
Avg Nucleus Perimeter (μm)	34.6	2.1	34.1	1.8	0.805	
Avg Nucleus Roundness	0.7	0.005	0.7	0.006	0.001**	−2.309
Area Analyzed (μm^2^)	5E+ 07	2E+ 07	5E+ 07	2E+ 07	0.646	

Quality control parameters were quantified from the regions of interest (ROIs) used in the multiplex analysis presented in Table 3 and Figs. 4 and 5. The parameters of normal (*n* = 7) and idiopathic pulmonary fibrosis (IPF, *n* = 7) lung tissue was compared using Mann-Whitney U test. Effect size (Cohen’s D) was calculated for comparisons that were significantly different (*p < 0.05, **p < 0.01). Avg, average

Multiplex quality control variables for each of the panels are detailed in Table 3. There were no differences in cell area, cytoplasm area, nucleus area, nucleus perimeter or total area analysed between NDC and IPF tissues. Average nucleus roundness was smaller statistically (less round) in IPF compared to NDC tissues but this parameter does not affect the number of counted cells and is likely a reflection of minor cell compression in fibrotic tissue [16].

### Statistics

Prism Software Version 7 (GraphPad Software Inc., USA) to compare IPF and NDC data. For flow cytometric analysis, between 2-group comparisons were performed using unpaired t-test with Welch’s correction. For tissue brightfield histological analysis between 3-group comparisons was made using one-way ANOVA with Dunnett’s multiple comparisons test. For tissue multiplex histological analysis between 2-group comparisons was made using Mann-Whitney U-test.

## Results

The first aim of this study was to investigate the utility of circulating CXCR4^+^ PMBCs as a potential biomarker in idiopathic pulmonary fibrosis (IPF). The second aim was to then quantify and characterize two populations of interstitial CXCR4^+^ cells in lung tissue of patients with end-stage pulmonary fibrosis, including IPF.

### The percentage of circulating CXCR4^+^ cells is lower in IPF patients compared to NDC donors but the proportion of CXCR4^+^ cells does not correlate with clinical parameters

**Fig. 1:**
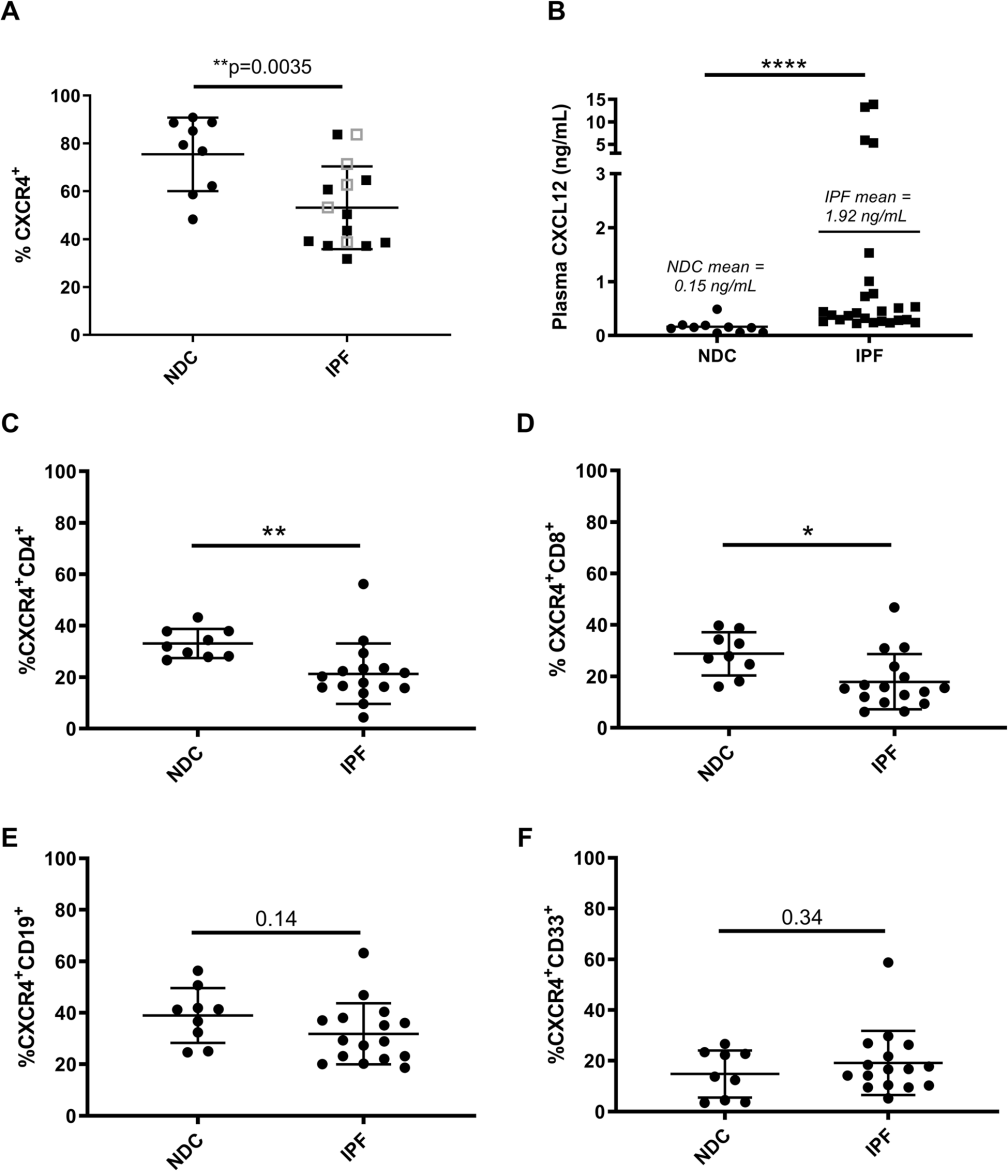
Percentage of CXCR4^+^ cells in whole blood is lower in IPF. **a** PBMCs were isolated from whole blood of 16 patients with idiopathic pulmonary fibrosis (IPF) and 9 age-matched, non-diseased control (NDC) donors where all samples analysed contained more than 5000 CXCR4^+^ cells. **b** Plasma was isolated from whole blood of 20 patients with idiopathic pulmonary fibrosis (IPF) and 10 age-matched, non-diseased control (NDC) donors. CXCL12 levels were measured by ELISA. **c** The percentage of CXCR4^+^ (%CXCR4^+^) cells was lower in patients with IPF compared to NDC donors. There was no difference in %CXCR4^+^ between IPF patients on anti-fibrotic therapy (pirfenidone or nintedanib *n* = 10, ■) or those who were not (*n* = 6, □) at the time of blood sampling. The percentage of (**d**) CXCR4^+^/CD4^+^ (**e**) CXCR4^+^/CD8^+^ cells were lower in IPF compared to NDC but there was no difference in the percentage of (**f**) CXCR4^+^/CD19^+^ and (**g**) CXCR4^+^/CD33^+^ cells between the two groups. Data presented as mean and standard deviation. Mann-Whitney U test, *p < 0.05, **p < 0.01, ****p < 0.0001

The percentage of all CXCR4^+^ cells in 16 IPF patients was significantly lower compared to 9 NDC (mean 75% vs 53%, *p* = 0.004) (Fig. 1a), with fewer CXCR4^+^/CD4^+^ and CXCR4^+^/CD8^+^ cells in IPF patients than NDCs (Fig. 1c, d). In contrast, there was no difference in the percentage of CXCR4^+^/CD19^+^ (B-cell origin) or CXCR4^+^/CD33^+^ (early myeloid origin) cells (Fig. 1e, f) between the two groups. In addition, there was no observed difference in %CXCR4^+^ between IPF patients on anti-fibrotic therapy (pirfenidone or nintedanib *n* = 10) or those who were not (*n* = 6). There was no correlation between %CXCR4^+^ cells and forced vital capacity (FVC, % predicted), transfer factor of carbon monoxide (TLCO, % predicted), or 6-min walk test (6MWT, m) in patients with IPF (Additional Figure 6).

### Plasma CXCL12 is increased in IPF

CXCL12 plasma concentrations in IPF patients were significantly higher compared with the NDC group (mean 0.15 vs 1.92 ng/mL, *p* < 0.0001) (Fig. 1b). In 5 IPF patients who were followed longitudinally, CXCL12 levels remained relative stable over several months (Additional Figure 7).

### Two populations of CXCR4^+^ cells are seen in peripheral lung tissue

**Fig. 2:**
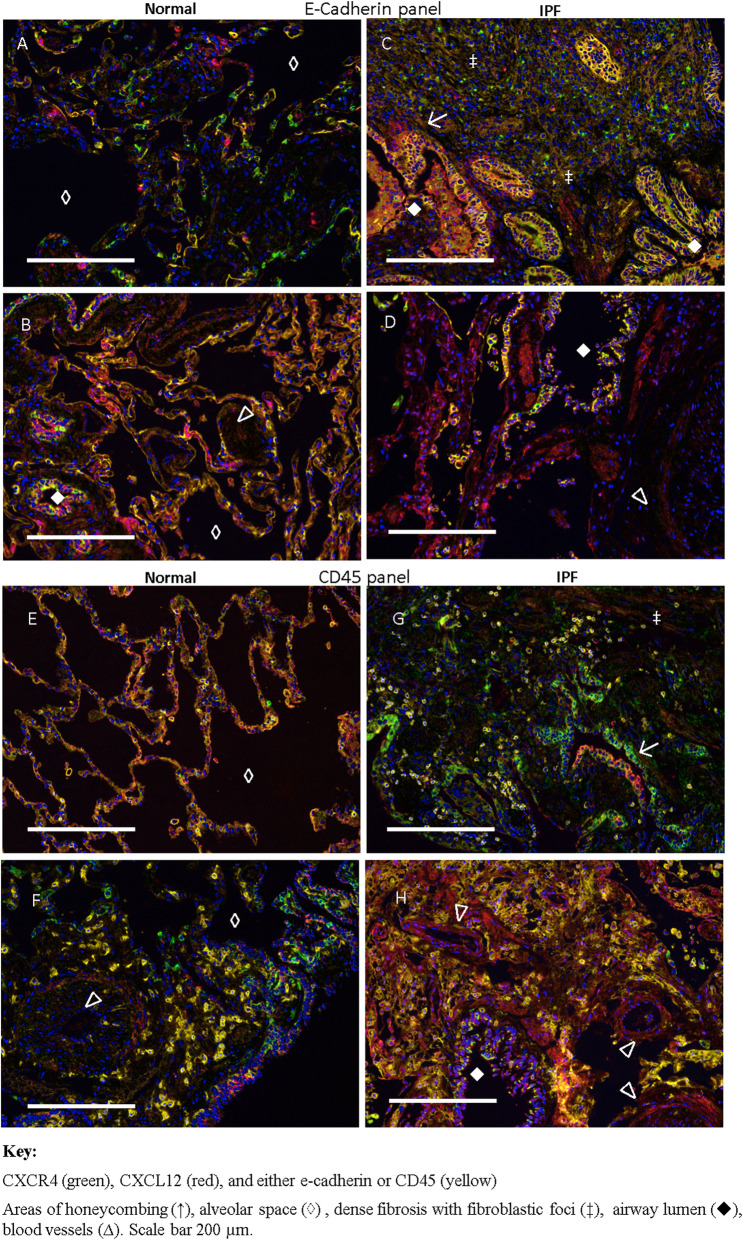
Two populations of CXCR4^+^ cells are seen in peripheral lung tissue. Multiplex immunohistochemistry was used to identify two populations of CXCR4-expressing cells in lung tissue from 7 patients with IPF and 7 non-diseased control (NDC) donors. Tissue was stained simultaneously for CXCR4 (green), CXCL12 (red), and either e-cadherin or CD45 (yellow) to identify epithelial cells and myeloid cells respectively. Cell nuclei was counterstained with Hoechst dye. In the e-cadherin panel (**a**-**d**) CXCR4^+^/e-cadherin^+^ cells can be seen in areas of honeycombing (↑) that may or may not also express CXCL12. In NDC tissue (**a**-**b**) the occasional circulating cell can be seen in the alveolar space (◊) but in IPF tissue (**c**-**d**) this has been replaced by dense fibrosis with fibroblastic foci (‡). In the CD45 panel (**e**-**h**) CXCR4^+^ cells can be seen surrounding the airway lumen (♦) in IPF tissue but there is a lack of CXCR4^+^ cells in blood vessels (∆). Scale bar 200 μm

CXCR4^+^ cells of epithelial origin were identified by co-expression of e-cadherin and cells of myeloid origin were identified by CD45. Figure 2 shows representative images of tissues taken from each of the 14 individuals included in the multiplex IHC study.

In tissue stained sequentially for CXCR4, e-cadherin and CXCL12 (“e-cadherin panel”), samples from NDC donors showed that CXCR4 expression localised predominantly to the airway epithelium. (Fig. 2a-b). NDC donors had almost no expression of CXCR4 although the rare CXCR4^+^ (but e-cadherin^−^ and CXC12^−^) could be seen within the alveolar space (◊).Some NDC donors had higher numbers of CXCR4 expressing cells and few CXCL12^+^ cells (Fig. 2a) compared to others that expressed higher levels of CXCL12 (Fig. 2b). In tissue from patients with IPF, strong CXCR4 expression was observed in hyperplastic epithelium and honeycomb cysts (↑) (Fig. 2c). Strong CXCL12 expression was also seen in cells within the airway lumen (♦) (Fig. 2d), smooth muscle bundles next to the CXCR4^+^ airway (Fig. 2d) and within fibroblast foci (‡) (Fig. 2c).

In NDC tissue stained sequentially for CXCR4, CD45 and CXCL12 (“CD45 panel”), the CD45^+^ cells that could be seen did not commonly express CXCR4 (Fig. 2e). It appeared that CD45^+^ cells surrounding small blood vessels (∆) in NDC tissue did not express CXCR4 although other nearby cells clearly did (Fig. 2f). In patients with IPF, some infiltrating CD45^+^ cells expressed high levels of CXCL12 but not CXCR4 in areas of honeycombing (Fig. 2g). IPF patients had relatively higher interstitial expression of CXCL12, particularly in blood vessel walls, but those cells did not show simultaneous expression of CXCR4 (Fig. 2h).

### Various phenotypes of CXCR4^+^ cells are observed in lung tissue

**Fig. 3:**
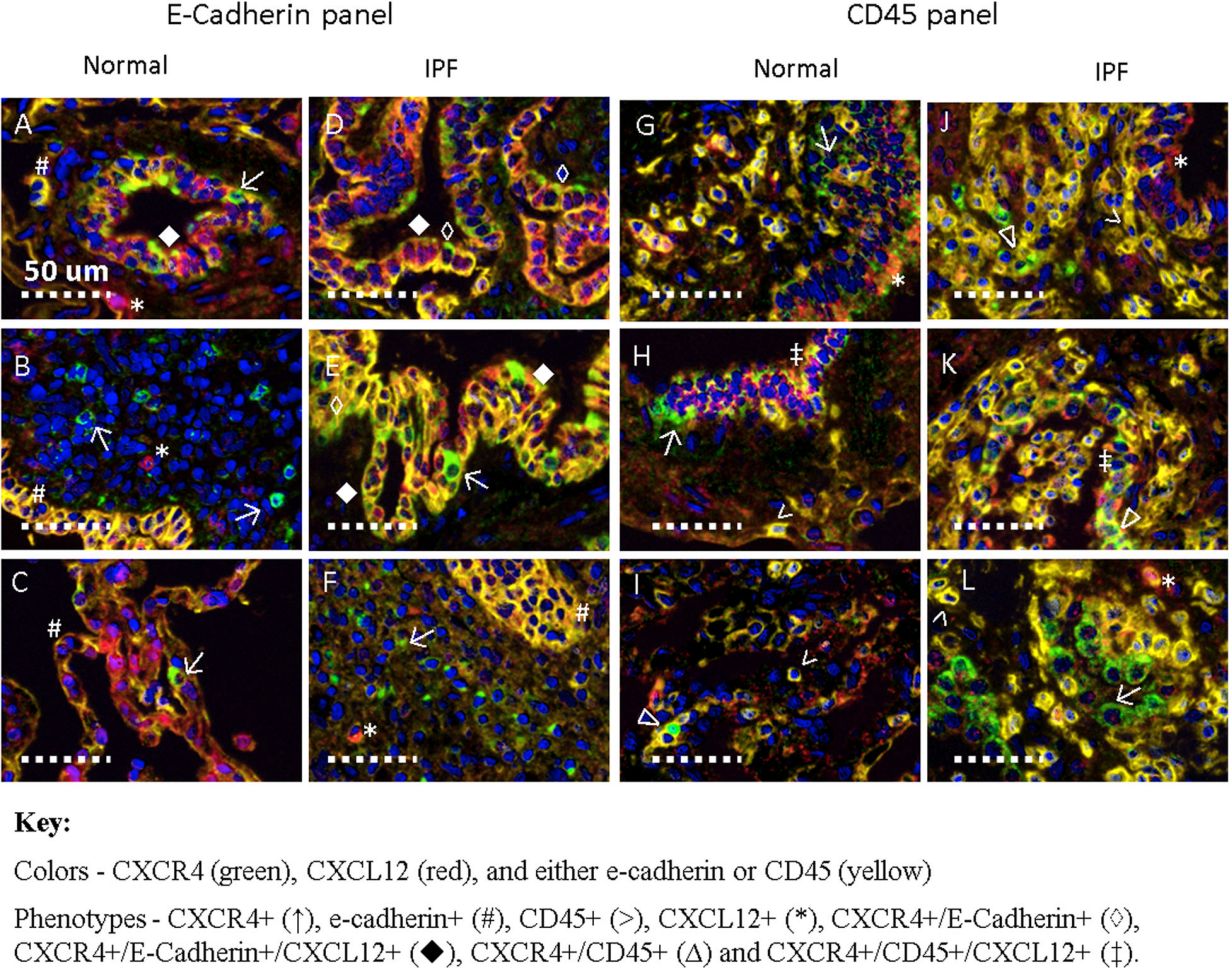
Various phenotypes of CXCR4^+^ cells are observed in lung tissue. Representative images of lung tissue from 3 patients with IPF and 3 non-diseased control (NDC) donors. Tissue was stained simultaneously for CXCR4 (green), CXCL12 (red), and either e-cadherin or CD45 (yellow) to identify epithelial cells and myeloid cells respectively. Cell nuclei was counterstained with Hoechst dye. Eight cell phenotypes were observed in NDC (**a**-**c**, **g**-**i**) and IPF (**d**-**f**, **j**-**l**) tissue. CXCR4^+^ (↑), e-cadherin^+^ (#), CD45^+^ (>), CXCL12^+^ (*), CXCR4^+^/E-Cadherin^+^ (◊), CXCR4^+^/E-Cadherin^+^/CXCL12^+^ (◆), CXCR4^+^/CD45^+^ (∆) and CXCR4^+^/CD45^+^/CXCL12^+^ (‡). Scale bar 50 μm

To illustrate the morphology and context of CXCR4^+^ cells in lung tissue, higher magnification shows the 8 main phenotypes of CXCR4^+^ cells that were quantified in this study (Fig. 3). From tissue stained with the e-cadherin panel, single positive CXCR4^+^ cells (↑) were observed in NDC tissue which had a clear epithelial morphology (Fig. 3a, c) or clearly did not (Fig. 3b). In IPF tissue, double positive CXCR4^+^/e-cadherin^+^ cells (◊) did not always simultaneously express CXCL12, however these triple positive cells were easily observable (♦) (Fig. 3d, e). Single positive CXCL12^+^ cells (*) were also observed that did not express CXCR4 or e-cadherin (Fig. 3f).

From NDC tissue stained with the CD45 panel, most CXCR4^+^ cells within the epithelium were not CD45^+^ (Fig. 3g, h) but the rare CXCR4^+^/CD45^+^ cell (∆) could be seen in the normal interstitium (Fig. 3i). These double positive CXCR4^+^/CD45^+^ cells were more evident in tissue from IPF patients (Fig. 3j). Triple positive CXCR4^+^/CD45^+^/CXCL12^+^ cells (‡) were only evident to any extent in IPF lung tissue (Fig. 3k) and there were also CXCR4^+^ cells which did not express CD45 or CXCL12 (Fig. 3l) that did not appear to be of epithelial morphology.

### Epithelial-origin CXCR4^+^ and myeloid-origin CXCR4^+^ cells are increased in IPF lung tissue

**Fig. 4:**
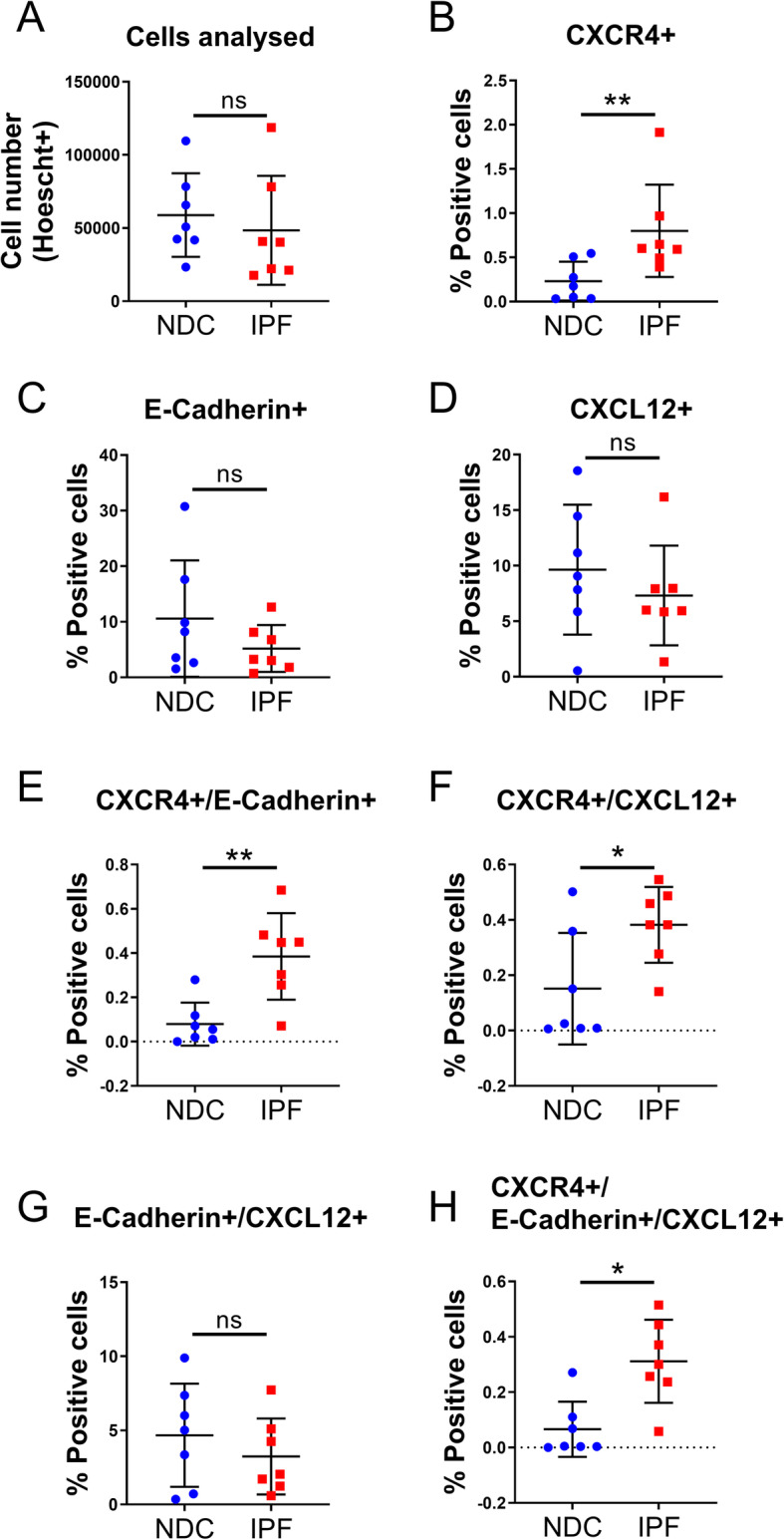
Epithelial CXCR4^+^ cells are increased in IPF lung tissue. Quantification of CXCR4^+^ phenotypes in lung tissue from 7 non-diseased control (NDC) donors and 7 patients with idiopathic pulmonary fibrosis (IPF) was performed using multiplex immunohistochemistry and analysed using automated image analysis (HALO). **a** The total number of cells used in the analysis was not different between NDC and IPF groups. **b** The percentage of single positive CXCR4 cells was increased in IPF compared to NDC. There is no difference in (**c**) the % e-cadherin^+^ cells or (**d**) the % CXCL12^+^ cells between the groups. (**e**) The percentage of double positive CXCR4/e-cadherin and (**f**) CXCR4^+^ /CXCL12^+^ cells were higher in IPF compared to NDC but there was no difference in the proportion of (**g**) e-cadherin^+^ /CXCL12^+^ cells. **h** The % of triple positive CXCR4^+^ /e-cadherin^+^ /CXCL12^+^ cells was increased in IPF compared to NDC. Data are presented as mean and standard deviation. Mann-Whitney U test, *p < 0.05, ** *p* < 0.01

Quantification data, including group means, standard deviation and effect size, from the multiplex immunohistochemistry are summarized in Table 4. To quantify the proportion of CXCR4 cells that also expressed e-cadherin and CXCL12, equal numbers of cells were counted in lung tissue from 7 NDC donors and 7 patients with IPF (Fig. 4a). The proportion of single positive CXCR4^+^ cells was increased in patients with IPF compared to NDC donors (effect size 1.494, *p* = 0.007) (Fig. 4b), but there was no difference in the proportion of cells expressing e-cadherin^+^ or CXCL12^+^ alone between the IPF and NDC groups (Fig. 4c, d). In IPF, there was an increase in the proportion of CXCR4^+^ cells that were of epithelial-origin (effect size 1.853, *p* = 0.007) (Fig. 4e) or were “activated” as they also expressed CXCL12 (effect size 1.349, *p* = 0.049) (Fig. 4f). However, there was no difference in epithelial cells expressing only e-cadherin and CXCL12 between IPF and NDC tissues (Fig. 4g). The proportion of CXCR4^+^/e-cadherin^+^/CXCL12^+^ cells was increased in IPF compared to NDC (effect size 1.815, *p* = 0.011).

**Table 4:** Summary of CXCR4 panel multiplex immunohistochemistry analysis

E-Cadherin Panel	Normal (*N* = 7)		IPF (*N* = 7)		Mann-Whitney U	Effect Size
	Mean1	SD	Mean2	SD	*p*-value	
Percentage “cells” = Hoechst+	30.69	13.6	28.91	11.5	0.209	
Single CXCR4+	0.23	0.2	0.80	0.5	0.007**	1.494
Single E-Cadherin+	10.59	10.5	5.20	3.9	0.318	
Single CXCL12+	9.64	5.9	7.32	5.8	0.456	
Double CXCR4 + E-Cadherin+	0.08	0.1	0.38	0.2	0.007**	1.853
Double CXCR4 + CXCL12+	0.15	0.2	0.38	0.1	0.049*	1.349
Double E-Cadherin+CXCL12+	4.67	3.5	3.24	2.4	0.620	
Triple CXCR4+ E-Cadherin+ CXCL12+	0.07	0.1	0.31	0.2	0.011*	1.815
Triple CXCR4+ E-Cadherin- CXCL12-	0.07	0.1	0.34	0.1	0.097	
Triple CXCR4+ E-Cadherin+ CXCL12-	0.01	0.0	0.07	0.1	0.073	
Triple CXCR4+ E-Cadherin- CXCL12+	0.09	0.2	0.07	0.2	0.383	
Triple CXCR4- E-Cadherin- CXCL12-	15.06	8.3	19.30	8.9	0.535	
Triple CXCR4- ECadherin- CXCL12+	4.88	4.9	4.00	5.1	0.535	
Triple CXCR4- E-Cadherin+ CXCL12+	4.61	3.4	2.93	3.4	0.535	
Triple CXCR4- E-Cadherin+ CXCL12-	5.90	7.8	1.88	7.4	0.456	
**CD45 Panel**	Normal (*N* = 7)		IPF (*N* = 7)		Mann-Whitney U	Effect Size
	Mean1	SD	Mean2	SD	*p*-value	
Percentage “cells” = Hoechst+	28.13	14.6	24.57	10.9	0.456	
Single CXCR4+	0.62	0.8	3.88	3.2	0.026*	1.378
Single CD45+	18.83	9.2	12.62	9.4	0.209	
Single CXCL12+	6.40	3.8	6.15	5.1	0.710	
Double CXCR4 + CD45+	0.17	0.1	0.78	0.8	0.026*	1.041
Double CXCR4 + CXCL12+	0.22	0.3	1.61	1.6	0.011*	1.186
Double CD45 + CXCL12+	5.28	3.0	3.49	3.9	0.209	
Triple CXCR4 + CD45 + CXCL12+	0.06	0.1	0.30	0.3	0.128	
Triple CXCR4+ CD45- CXCL12-	0.30	0.5	1.78	1.8	0.128	
Triple CXCR4+ CD45+ CXCL12-	0.11	0.1	0.48	0.2	0.097	
Triple CXCR4+ CD45- CXCL12+	0.16	0.2	1.32	0.7	0.011*	2.245
Triple CXCR4- CD45- CXCL12-	7.88	6.4	7.52	4.6	0.902	
Triple CXCR4- CD45- CXCL12+	0.96	0.9	1.34	0.9	> 0.9999	
Triple CXCR4- CD45+ CXCL12+	5.22	2.9	3.20	3.4	0.209	
Triple CXCR4- CD45+ CXCL12-	13.45	9.2	8.64	8.1	0.318	

Multiplex immunohistochemistry was performed, and cell phenotypes were quantified. The percentage of each cell phenotype in normal (*n* = 7) and idiopathic pulmonary fibrosis (IPF, *n* = 7) lung tissue was compared using Mann-Whitney U test. Effect size (Cohen’s D) was calculated for comparisons that were significantly different (*p < 0.05, **p < 0.01). Avg, average

**Fig. 5:**
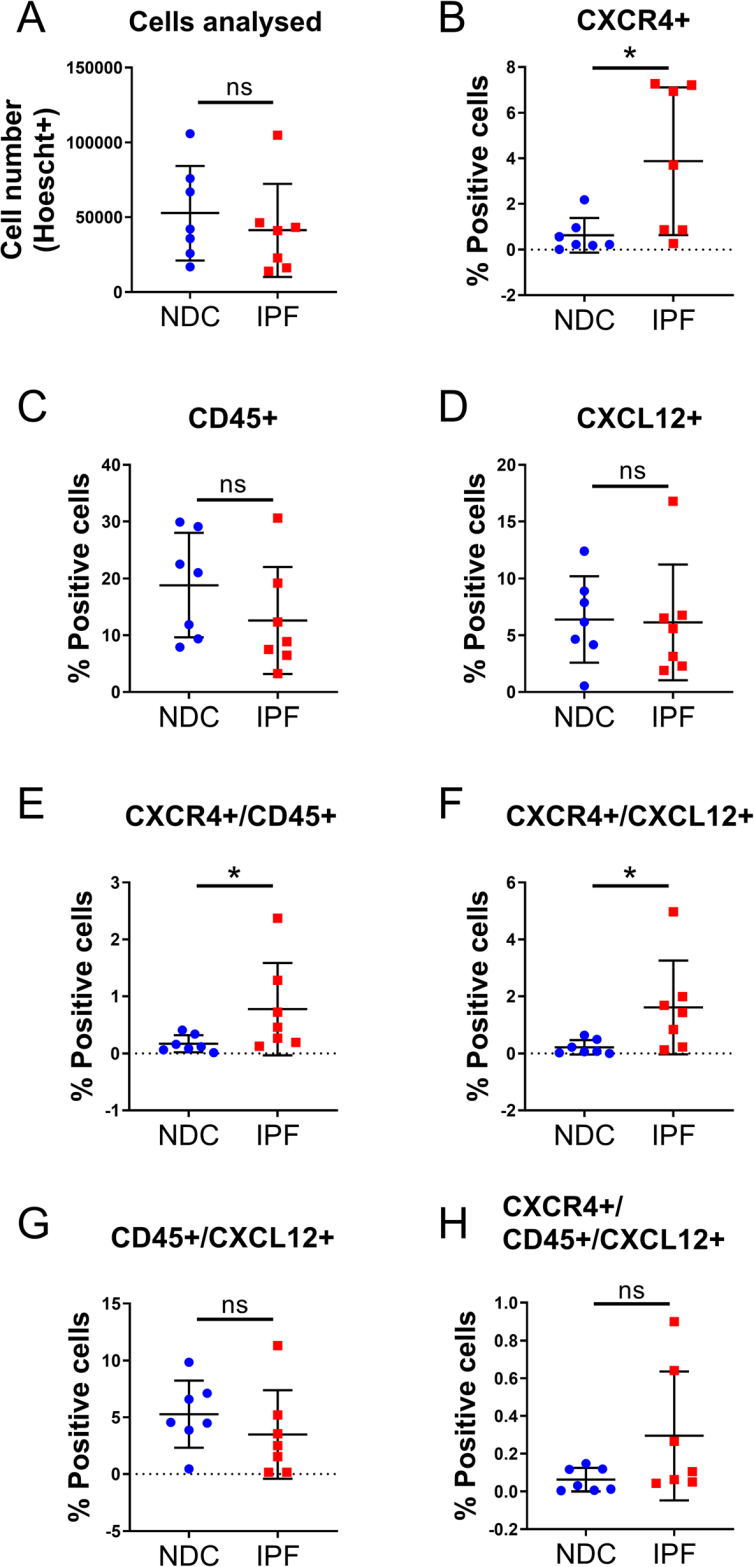
Myeloid CXCR4^+^ cells are increased in IPF lung tissue. Quantification of CXCR4^+^ phenotypes in lung tissue from 7 non-diseased control (NDC) donors and 7 patients with idiopathic pulmonary fibrosis (IPF) was performed using multiplex immunohistochemistry and analysed using automated image analysis (HALO). **a** The total number of cells used in the analysis was not different between NDC and IPF groups. **b** The percentage of single positive CXCR4 cells was increased in IPF compared to NDC. There is no difference in (**c**) the % CD45^+^ cells or (**d**) the % CXCL12^+^ cells between the groups. **e** The percentage of double positive CXCR4^+^/CD45^+^ and (**f**) CXCR4^+^/CXCL12^+^ cells were higher in IPF compared to NDC but there was no difference in the proportion of (**g**) CD45^+^/CXCL12^+^ cells or (**h**) triple positive CXCR4^+^/CD45^+^/CXCL12^+^ cells. Data are presented as mean and standard deviation. Mann-Whitney U test, *p < 0.05

To quantify the proportion of CXCR4 cells that also expressed CD45 and CXCL12, equal numbers of cells were counted in both groups (Fig. 5a). Mirroring data shown in Fig. 4, the proportion of single positive CXCR4 cells was increased in lung tissue from IPF compared to NDC (effect size 1.378, *p* = 0.026) (Fig. 5b) and there was no difference in the proportion of CD45^+^ (Fig. 5c) or CXCL12^+^ cells (Fig. 5d). In IPF, the proportion of CXCR4^+^ cells that were also CD45^+^ was increased compared to NDC (effect size 1.041, *p* = 0.0262) (Fig. 5e). Similar to our previous result, the proportion of CXCR4^+^ cells that were also CXCL12^+^ was increased in IPF compared to NDC (effect size 1.186, p = 0.011) (Fig. 5f). However, the proportion of cells that were CD45^+^/CXCL12^+^ or CXCR4^+^/CD45^+^/CXCL12^+^ was not different between the two groups (Fig. 5g, h).

Thus, CXCR4^+^ cells in IPF lungs appeared to derive from both an epithelial and a myeloid origin.

### Brightfield immunohistochemistry of lung tissue from patients with end-stage fibrotic interstitial lung disease (ILD) and normal donors

**Fig. 6:**
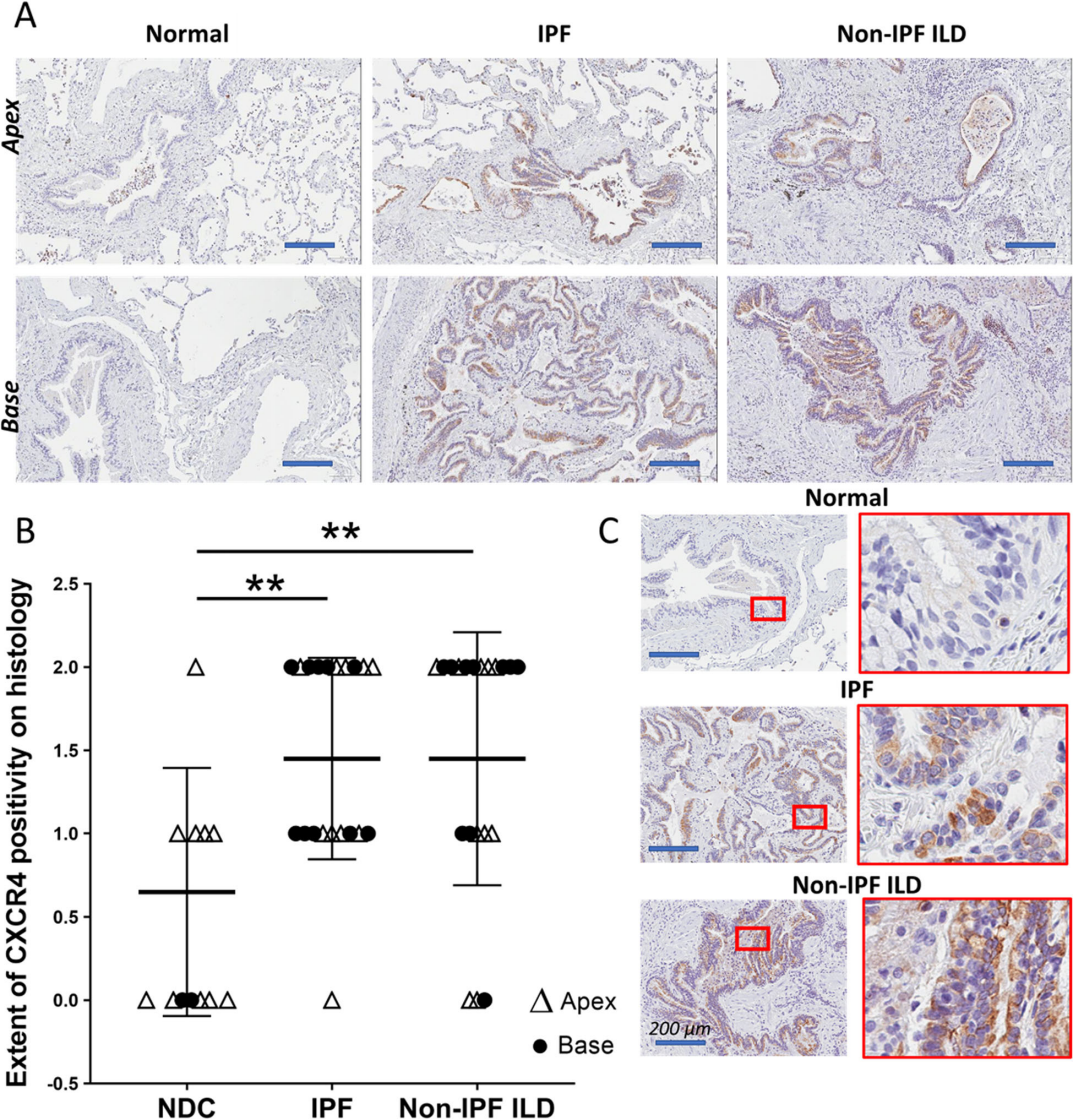
Brightfield immunohistochemistry of lung tissue from patients with end-stage fibrotic interstitial lung disease (ILD) and normal donors. Lung tissue was sampled from 10 patients with idiopathic pulmonary fibrosis (IPF), 8 patients with non-IPF ILD (2 non-specific interstitial pneumonia, 4 hypersensitivity pneumonitis, 2 connective tissue disease associated ILD and 10 non-diseased control (NDC) normal donors. Brightfield immunohistochemistry was used to examine CXCR4 expression in lung tissue with cell nuclei counterstained with Mayer’s hematoxylin. **a** Minimal CXCR4 expression is seen in NDC lung tissue. Strong CXCR4 staining is seen in tissue taken from lung apices and bases in both IPF and non-IPF ILD patients. Scale bar 200 μm (**b**) CXCR4 expression was semi-quantified in 60 samples of lung tissue from lung apex (∆) and lung bases (•). CXCR4 is increased in IPF and non-IPF ILD compared to NDC donors. **c** Higher magnification of base-derived lung tissue shown in (A) demonstrates CXCR4 expression in distal airways localised to epithelium in IPF and non-IPF ILD. Scale bar 200 μm

Lung tissue sampled from the apex and base of 10 patients with IPF, 8 patients with non-IPF, end-stage ILD and 10 NDC donors showed that under normal conditions, CXCR4 expression in minimal. In fibrotic conditions, CXCR4 expression is easily observed in epithelium of tissue from patients with end-stage ILD as well as IPF (Fig. 6a). Semi-quantification of CXCR4 staining demonstrated that CXCR4 expression is also elevated in the epithelium of patients with end-stage ILD compared to NDC donors (Fig. 6b). CXCR4 expression can be observed localising to fibrotic epithelium in higher magnification of brightfield images (Fig. 6c).

## Discussion

The precise pathophysiological pathways that drive idiopathic pulmonary fibrosis (IPF) are yet to be fully understood. Earlier work suggests that the CXCR4 pathway may be a therapeutic target [17, 18]. We have identified two distinct populations of CXCR4^+^ cell, one of epithelial origin (CXCR4^+^/e-cadherin^+^) and one of myeloid origin (CXCR4^+^/CD45^+^), that were increased in lung tissue of patients with IPF compared to non-diseased control (NDC) donors. Furthermore, we now provide more evidence that dysregulation of CXCR4 expression is not unique to IPF and therefore our findings are relevant to patients with fibrotic ILDs as well.

Fibrocytes are thought to be one source of fibroblasts in IPF by differentiating into matrix-producing myofibroblasts [19] after trafficking into the lung from the circulation. One study reported that most circulating fibrocytes express CXCR4 [20] and importantly, fibrocytes are not found in the tissue of normal lungs [21], therefore, lower levels of CXCR4^+^ cells in IPF patient circulation compared to NDC donors was unexpected and out of line with the literature [22]. It was not previously known if monitoring circulating cells by using only CXCR4^+^ as an identifier could be a surrogate marker of active fibrosis. Our study suggests that there exists a normal circulating level of CXCR4^+^ cells in healthy individuals which may be reduced as a result of active fibrosis in the lung tissue. These cells may be migrating from the circulation to the interstitial tissue through a chemical gradient produced by CXCL12-expressing cells, such as fibroblasts, or via activation in the circulation through elevated plasma CXCL12 levels.

Plasma CXCL12 concentration is elevated in chronic hypersensitivity pneumonitis, another related fibrotic ILD, and may be epithelial cell-derived [23]. The previous studies that have observed increased numbers of CXCR4^+^ cells in fibrotic lung tissue have primarily focused on the fibrocyte subtype which express haemopoietic markers like CD45 and Collagen 1 in addition to CXCR4 [19–21, 24]. Relatively few studies have examined the contribution of CXCR4^+^ epithelial cells to pulmonary fibrosis [4, 25, 26]. During wound healing events, CXCR4 expression on alveolar type II (ATII) cells is important to drive epithelial cell migration and proliferation [4, 25, 27]. Both CXCR4 and CXCL12 expression is induced in ATII cells following lung injury [28] and both genes are regulated by a hypoxic microenvironment [29] such as in fibrotic tissue. Our study suggests that in the fibrotic lung, chronic injury and hypoxia drives constant upregulation of CXCR4 and CXCL12 in resident epithelial cells at the epithelial-alveolar interface, leading to overproliferation and migration of neighbouring ATII cells and driving alterations in the tissue structure.

The initiating event that leads to the formation of usual interstitial pneumonia (UIP) remains unknown but hyperplastic ATII cells are often seen at areas of active fibrosis in patients with fibrotic ILD [30] and there is some evidence to suggest that microscopic honeycomb cyst formation has a distal airway origin driven by dysregulation of the mucin gene MUC5B [14]. Recently, Chen and colleagues demonstrated the therapeutic potential of targeting honeycomb cyst formation via MUC5B inhibition in IPF [31]. Similarly, our study suggests that targeting epithelial CXCR4^+^ cells may prevent honeycomb cyst formation in IPF and other fibrotic ILDs that share the UIP pattern. It was outside the scope of this study to determine if there were differences in CXCR4 expression between IPF and the other fibrotic ILDs but it can be concluded that the UIP phenotype includes alveolar destruction involving epithelial CXCR4^+^ cells. Furthermore, these epithelial CXCR4^+^ cells, unlike their lymphoid counterparts, the CXCR4^+^CD45^+^ cells, also expressed CXCL12, indicating that the epithelium may be the primary site of fibrotic activity driven by mechanisms involving the CXCR4/CXCL12 axis in IPF. Future studies in a larger cohort are needed to determine the precise cell type and investigate the role of CXCR4^+^ cells that do not express either e-cadherin or CD45.

The limitation of our study was that the donors from whom PMBCs were obtained were not the same donors who had provided normal plasma. Furthermore, the IPF patients who provided whole blood were not the same patients from whom lung tissue was investigated. Therefore, it is difficult to determine how the quantity of CXCR4^+^ PBMCs relates to CXCL12 plasma levels in normal donors or to numbers of CXCR4^+^ cells in IPF lung tissue. Our study did not show any differences in CXCR4^+^/CD19^+^ (B-cell origin) or CXCR4^+^/CD33^+^ (early myeloid origin) cells, and therefore further investigation with a larger panel of surface markers may be necessary to delineate whether other circulating CXCR4^+^ cells are clinically relevant in IPF.

## Conclusion

While the number of circulating CXCR4^+^ cells alone may not be a sufficient marker of disease progression in IPF, this study adds to the body of evidence that CXCR4/CXCL12 axis is an important mechanism for development of fibrosis. This data suggests that targeting of epithelial CXCR4 may be an appropriate therapeutic strategy for pulmonary fibrosis, particularly in patients with IPF and potentially those with non-IPF ILD. Long term safety data for the first generation of CXCR4 antagonists used for patients with acute myeloid leukemia, where a high CXCR4 expression is linked to poor progression [11], demonstrates that CXCR4 inhibition is a well-tolerated approach. Further studies are required to determine what role epithelial-CXCR4^+^ cells have in the progression of IPF.

## Supplementary information

**Additional file 1: Figure 1.** Colour deconvolution for multiplex analysis (PNG file). (A) Representative multiplex 20x image containing signals from 4 spectrally distinct fluorophores which are then separated for downstream image analysis. (B) Cell nuclei are stained with Hoechst (blue). (C) CXCR4 cells are stained with Opal520™ (green). (D) E-cadherin expressing cells are in yellow (Opal570™). (E) CXCL12 is red (Opal690™).

**Additional file 2: Figure 2.** Region of Interest (ROI) generation and exposure settings for multiplex analysis (TIFF file). (A) Representative image showing an IPF tissue section stained with panel 1 (e-cadherin panel) with automatically generated ROIs covering the entire section. (B) Representative image showing an IPF tissue section stained with panel 2 (CD45 panel) with automatically generated ROIs covering the entire tissue section. Table details the emission and excitation wavelengths of each of the OPAL dyes used in the study as well as the exposure settings used on the Vectra 3 fluorescent scanner.

**Additional file 3: Figure 3.** Thresholds for positive cell quantification in multiplex analysis.

**Additional file 4: Figure 4.** Cell phenotype quantification in e-cadherin panel (TIFF file). Representative analysis performed on a single 20x image of tissue from (A-D) a non-diseased control (NDC) donor and (E-F) a patient with idiopathic pulmonary fibrosis (IPF). Cells are automatically identified based on expression of Hoechst (blue) and morphological characteristics such as nuclear perimeter and roundness. Thresholds for each of the phenotype markers were set manually and positive cells were automatically identified. (A) Small airway section showing normal alveolar space (◊) with mild infiltrate within the airway lumen (♦). (B) CXCR4^+^ cells (green) are absent. (C) E-cadherin^+^ cells (yellow) can be seen in the airway epithelium and lining alveolar spaces. (D) CXCL12^+^ cells (red) are present in the airway epithelium. (E) Distal parenchymal tissue showing small airway (↑) and associated blood vessel (∆) within densely fibrotic tissue. (F) CXCR4^+^ cells are seen within the small airway and express both (G) e-cadherin and (H) CXCL12. Scale bar 200 μm.

**Additional file 5: Figure 5.** Cell phenotype quantification in CD45 panel (TIFF file). Representative analysis performed on a single 20x image of tissue from (A-D) a non-diseased control (NDC) donor and (E-F) a patient with idiopathic pulmonary fibrosis (IPF). Cells are automatically identified based on expression of Hoechst (blue) and morphological characteristics such as nuclear perimeter and roundness. Thresholds for each of the phenotype markers were set manually and positive cells were automatically identified. (A) Small airway section showing normal alveolar space (◊) and airway lumen (♦). (B) CXCR4^+^ cells (green) are only seen in the epithelium and (C) there is mild inflammation consisting of CD45^+^ cells (yellow) in subepithelial interstitial tissue. (D) CXCL12^+^ cells (red) are found in the alveolar walls but not in the airway. (E) In IPF tissue where alveolar space still remains, microscopic capillaries (∆) can be seen. (F) Few CXCR4^+^ cells can be observed within thickened alveolar septa and near capillaries. (G) Mild inflammation is also a feature in IPF as CD45^+^ cells are seen throughout interstitial tissue. (H) CXCL12^+^ cells outnumber CXCR4^+^ cells in IPF. Scale bar 200 μm.

**Additional file 6: Figure 6.** Correlation between clinical parameters and %CXCR4^+^ cells in whole blood of patients with IPF (PNG file). Numbers of CXCR4^+^ cells were quantified by flow cytometry in 20 patients with idiopathic pulmonary fibrosis (IPF) and clinical variables (forced vital capacity, FVC and transfer factor of carbon monoxide, TLCO) and 6-min walk test (6MWT) distance was collected. The association between percentage of CXCR4^+^ cells and lung function or 6MWT was analysed by Spearman’s rank correlation co-efficient.

**Additional file 7: Figure 7.** Plasma CXCL12 stable over time (PNG file). In 5 patients with idiopathic pulmonary fibrosis who had repeated bloods drawn over several months, there was no change in plasma CXCL12 level. Data are presented as mean and standard deviation.

## Data Availability

The datasets used and/or analysed during the current study are available from the corresponding author on reasonable request.

## Abbreviations

ATII: Alveolar Type II
CTD: Connective tissue disease
CXCL12: C-X-C motif chemokine 12
CXCR4: C-X-C chemokine receptor type 4
HP: Hypersensitivity pneumonitis
ILD: Interstitial lung disease
IPF: Idiopathic pulmonary fibrosis
NDC: Non-diseased control
PBMC: Peripheral blood mononuclear cell
SDF-1: Stromal cell-derived factor 1
UIP: Usual interstitial pneumonia

## References

[1] Jo HE, Troy LK, Keir G, Chambers DC, Holland A, Goh N, Wilsher M, de Boer S, Moodley Y, Grainge C, Whitford H, Chapman S, Reynolds PN, Glaspole I, Beatson D, Jones L, Hopkins P, Corte TJ (2017). Treatment of idiopathic pulmonary fibrosis in Australia and New Zealand: a position statement from the Thoracic Society of Australia and new Zealand and the Lung Foundation Australia. Respirology..

[2] Olson AL, Gifford AH, Inase N, Fernández Pérez ER, Suda T (2018). The epidemiology of idiopathic pulmonary fibrosis and interstitial lung diseases at risk of a progressive-fibrosing phenotype. Eur Respir Rev.

[3] Wolters PJ, Collard HR, Jones KD (2014). Pathogenesis of idiopathic pulmonary fibrosis. Annu Rev Pathol.

[4] Murdoch C, Monk PN, Finn A (1999). Functional expression of chemokine receptor CXCR4 on human epithelial cells. Immunology..

[5] Rankin SM (2012). Chemokines and adult bone marrow stem cells. Immunol Lett.

[6] Xu J, Mora A, Shim H, Stecenko A, Brigham KL, Rojas M (2007). Role of the SDF-1/CXCR4 axis in the pathogenesis of lung injury and fibrosis. Am J Respir Cell Mol Biol.

[7] Mehrad B, Burdick MD, Zisman DA, Keane MP, Belperio JA, Strieter RM (2007). Circulating peripheral blood fibrocytes in human fibrotic interstitial lung disease. Biochem Biophys Res Commun.

[8] Griffiths K, Habiel DM, Jaffar J, Binder U, Darby WG, Hosking CG, Skerra A, Westall GP, Hogaboam CM, Foley M (2018). Anti-fibrotic effects of CXCR4-targeting i-body AD-114 in preclinical models of pulmonary fibrosis. Sci Rep.

[9] Gundavarapu S, Mishra NC, Singh SP, Langley RJ, Saeed AI, Feghali-Bostwick CA, McIntosh JM, Hutt J, Hegde R, Buch S, Sopori ML (2013). HIV gp120 induces mucus formation in human bronchial epithelial cells through CXCR4/α7-nicotinic acetylcholine receptors. PLoS One.

[10] Wagner PL, Hyjek E, Vazquez MF, Meherally D, Liu YF, Chadwick PA, Rengifo T, Sica GL, Port JL, Lee PC, Paul S, Altorki NK, Saqi A (2009). CXCL12 and CXCR4 in adenocarcinoma of the lung: association with metastasis and survival. J Thorac Cardiovasc Surg.

[11] Mannelli F, Cutini I, Gianfaldoni G, Bencini S, Scappini B, Pancani F, Ponziani V, Bonetti MI, Biagiotti C, Longo G, Bosi A (2014). CXCR4 expression accounts for clinical phenotype and outcome in acute myeloid leukemia. Cytometry B Clin Cytom.

[12] Gomperts BN, Strieter RM (2007). Fibrocytes in lung disease. J Leukoc Biol.

[13] Flaherty KR, Thwaite EL, Kazerooni EA, Gross BH, Toews GB, Colby TV, Travis WD, Mumford JA, Murray S, Flint A, Lynch JP, Martinez FJ (2003). Radiological versus histological diagnosis in UIP and NSIP: survival implications. Thorax.

[14] Seibold MA, Smith RW, Urbanek C, Groshong SD, Cosgrove GP, Brown KK, Schwarz MI, Schwartz DA, Reynolds SD (2013). The idiopathic pulmonary fibrosis honeycomb cyst contains a mucocilary pseudostratified epithelium. PLoS One.

[15] Prasad JD, Mahar A, Bleasel J, Ellis SJ, Chambers DC, Lake F, Hopkins PMA, Corte TJ, Allan H, Glaspole IN (2017). The interstitial lung disease multidisciplinary meeting: a position statement from the Thoracic Society of Australia and new Zealand and the Lung Foundation Australia. Respirology..

[16] Mitruț R, Pirici D, Stepan AE, Mărgăritescu C, Simionescu CE, Kesse AM, Militaru C (2019). Histopathological and morphometric study of fibrosis and nuclear Pleomorphism in dilated cardiomyopathy. Curr Health Sci J.

[17] White ES, Borok Z, Brown KK, Eickelberg O, Guenther A, Jenkins RG, Kolb M, Martinez FJ, Roman J, Sime P (2016). An American Thoracic Society official research statement: future directions in lung fibrosis research. Am J Respir Crit Care Med.

[18] Kreuter M, Bonella F, Wijsenbeek M, Maher TM, Spagnolo P (2015). Pharmacological treatment of idiopathic pulmonary fibrosis: current approaches, unsolved issues, and future perspectives. Biomed Res Int.

[19] Phillips RJ, Burdick MD, Hong K, Lutz MA, Murray LA, Xue YY, Belperio JA, Keane MP, Strieter RM (2004). Circulating fibrocytes traffic to the lungs in response to CXCL12 and mediate fibrosis. J Clin Invest.

[20] Mehrad B, Burdick MD, Strieter RM (2009). Fibrocyte CXCR4 regulation as a therapeutic target in pulmonary fibrosis. Int J Biochem Cell Biol.

[21] Andersson-Sjoland A, de Alba CG, Nihlberg K, Becerril C, Ramirez R, Pardo A, Westergren-Thorsson G, Selman M (2008). Fibrocytes are a potential source of lung fibroblasts in idiopathic pulmonary fibrosis. Int J Biochem Cell Biol.

[22] Moeller A, Gilpin SE, Ask K, Cox G, Cook D, Gauldie J, Margetts PJ, Farkas L, Dobranowski J, Boylan C, O'Byrne PM, Strieter RM, Kolb M (2009). Circulating fibrocytes are an indicator of poor prognosis in idiopathic pulmonary fibrosis. Am J Respir Crit Care Med.

[23] Garcia de Alba C, Buendia-Roldan I, Salgado A, Becerril C, Ramirez R, Gonzalez Y, Checa M, Navarro C, Ruiz V, Pardo A, Selman M (2015). Fibrocytes contribute to inflammation and fibrosis in chronic hypersensitivity pneumonitis through paracrine effects. Am J Respir Crit Care Med.

[24] Antoniou KM, Soufla G, Lymbouridou R, Economidou F, Lasithiotaki I, Manousakis M, Drositis I, Spandidos DA, Siafakas NM (2010). Expression analysis of angiogenic growth factors and biological axis CXCL12/CXCR4 axis in idiopathic pulmonary fibrosis. Connect Tissue Res.

[25] Ghosh MC, Makena PS, Gorantla V, Sinclair SE, Waters CM (2012). CXCR4 regulates migration of lung alveolar epithelial cells through activation of Rac1 and matrix metalloproteinase-2. Am J Physiol Lung Cell Mol Physiol.

[26] Shimizu Y, Dobashi K, Endou K, Ono A, Yanagitani N, Utsugi M, Sano T, Ishizuka T, Shimizu K, Tanaka S, Mori M (2010). Decreased interstitial FOXP3(+) lymphocytes in usual interstitial pneumonia with discrepancy of CXCL12/CXCR4 axis. Int J Immunopathol Pharmacol.

[27] Ghosh MC, Makena PS, Kennedy J, Teng B, Luellen C, Sinclair SE, Waters CM (2017). A heteromeric molecular complex regulates the migration of lung alveolar epithelial cells during wound healing. Sci Rep.

[28] McClendon J, Jansing NL, Redente EF, Gandjeva A, Ito Y, Colgan SP, Ahmad A, Riches DWH, Chapman HA, Mason RJ, Tuder RM, Zemans RL (2017). Hypoxia-inducible factor 1alpha signaling promotes repair of the alveolar epithelium after acute lung injury. Am J Pathol.

[29] Kang N, Choi SY, Kim BN, Yeo CD, Park CK, Kim YK, Kim TJ, Lee SB, Lee SH, Park JY, Park MS, Yim HW, Kim SJ (2019). Hypoxia-induced cancer stemness acquisition is associated with CXCR4 activation by its aberrant promoter demethylation. BMC Cancer.

[30] White ES, Lazar MH, Thannickal VJ (2003). Pathogenetic mechanisms in usual interstitial pneumonia/idiopathic pulmonary fibrosis. J Pathol.

[31] Chen G, Ribeiro CMP, Sun L, Okuda K, Kato T, Gilmore RC, Martino MB, Dang H, Abzhanova A, Lin JM, Hull-Ryde EA, Volmer AS, Randell SH, Livraghi-Butrico A, Deng Y, Scherer PE, Stripp BR, O'Neal WK, Boucher RC (2019). XBP1S regulates MUC5B in a promoter variant-dependent pathway in IPF airway epithelia. Am J Respir Crit Care Med.

