# Supplementary figures and images for: CXCR4+ cells are increased in lung tissue of patients with idiopathic pulmonary fibrosis
Source: Respir Res. 2020 Aug 26;21:221. doi: 10.1186/s12931-020-01467-0 (PMC7449054; doi:10.1186/s12931-020-01467-0)

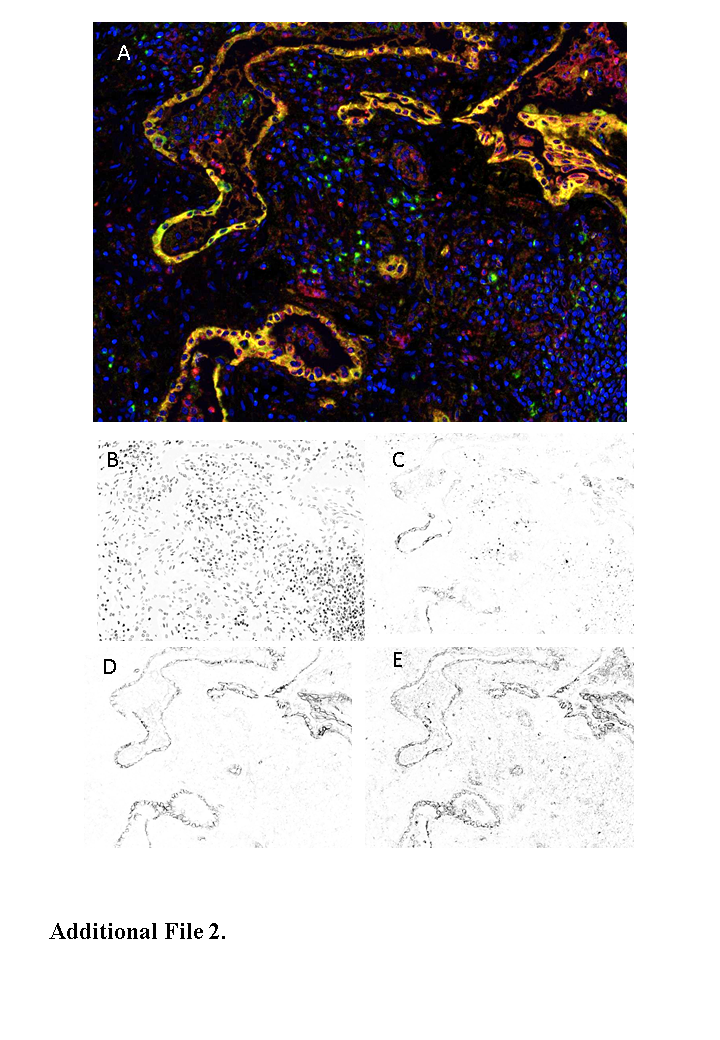

Supplement: Supplementary file 1 — Additional file 1: Figure 1. Colour deconvolution for multiplex analysis (PNG file). (A) Representative multiplex 20x image containing signals from 4 spectrally distinct fluorophores which are then separated for downstream image analysis. (B) Cell nuclei are stained with Hoechst (blue). (C) CXCR4 cells are stained with Opal520™ (green). (D) E-cadherin expressing cells are in yellow (Opal570™). (E) CXCL12 is red (Opal690™). [file 12931_2020_1467_MOESM1_ESM.png]

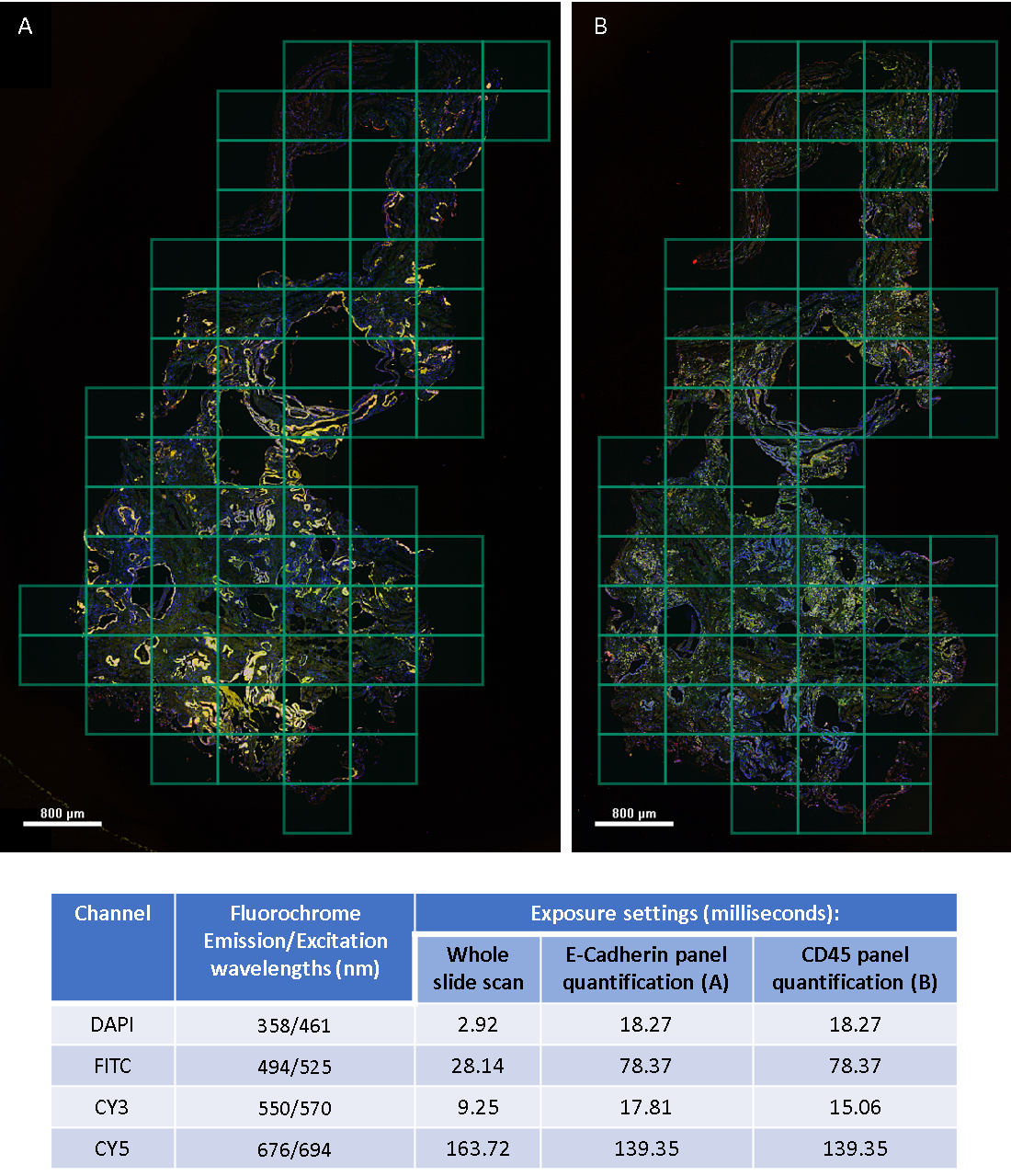

Supplement: Supplementary file 2 — Additional file 2: Figure 2. Region of Interest (ROI) generation and exposure settings for multiplex analysis (TIFF file). (A) Representative image showing an IPF tissue section stained with panel 1 (e-cadherin panel) with automatically generated ROIs covering the entire section. (B) Representative image showing an IPF tissue section stained with panel 2 (CD45 panel) with automatically generated ROIs covering the entire tissue section. Table details the emission and excitation wavelengths of each of the OPAL dyes used in the study as well as the exposure settings used on the Vectra 3 fluorescent scanner. [file 12931_2020_1467_MOESM2_ESM.tif]

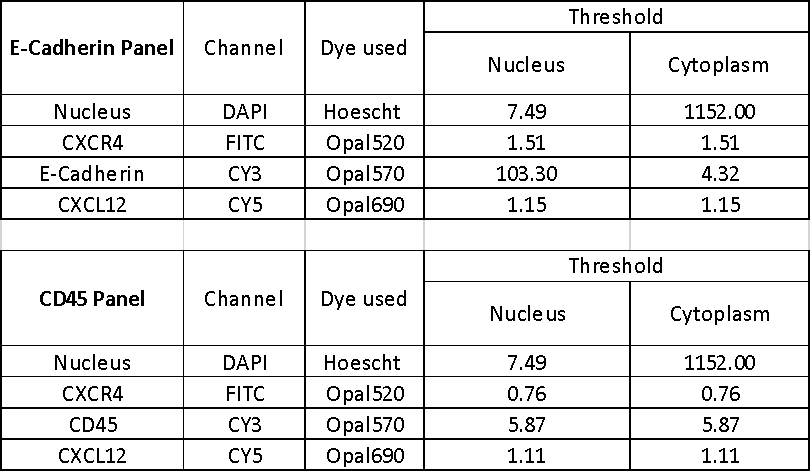

Supplement: Supplementary file 3 — Additional file 3: Figure 3. Thresholds for positive cell quantification in multiplex analysis. [file 12931_2020_1467_MOESM3_ESM.tif]

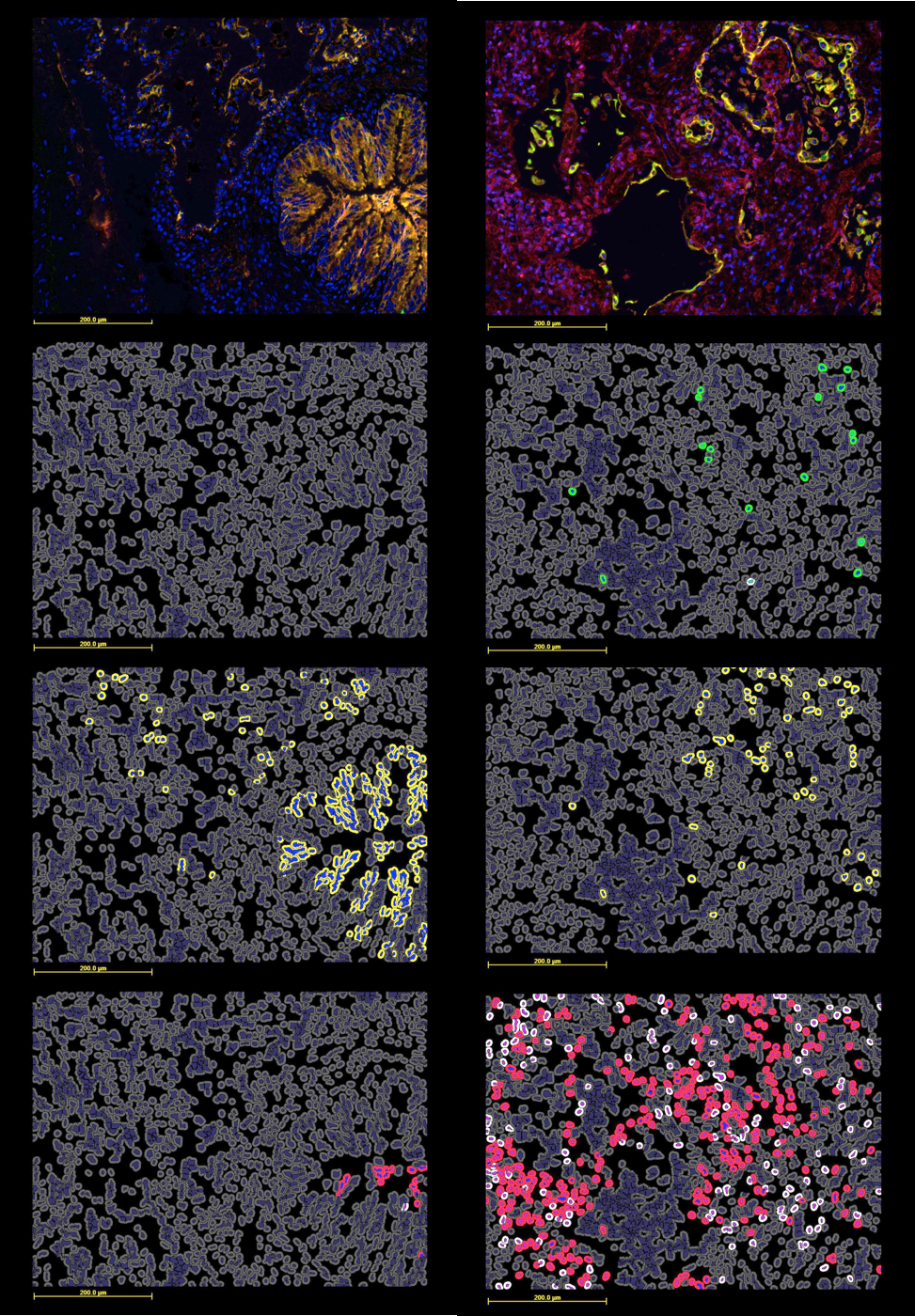

Supplement: Supplementary file 4 — Additional file 4: Figure 4. Cell phenotype quantification in e-cadherin panel (TIFF file). Representative analysis performed on a single 20x image of tissue from (A-D) a non-diseased control (NDC) donor and (E-F) a patient with idiopathic pulmonary fibrosis (IPF). Cells are automatically identified based on expression of Hoechst (blue) and morphological characteristics such as nuclear perimeter and roundness. Thresholds for each of the phenotype markers were set manually and positive cells were automatically identified. (A) Small airway section showing normal alveolar space (◊) with mild infiltrate within the airway lumen (♦). (B) CXCR4+ cells (green) are absent. (C) E-cadherin+ cells (yellow) can be seen in the airway epithelium and lining alveolar spaces. (D) CXCL12+ cells (red) are present in the airway epithelium. (E) Distal parenchymal tissue showing small airway (↑) and associated blood vessel (∆) within densely fibrotic tissue. (F) CXCR4+ cells are seen within the small airway and express both (G) e-cadherin and (H) CXCL12. Scale bar 200 μm. [file 12931_2020_1467_MOESM4_ESM.tif]

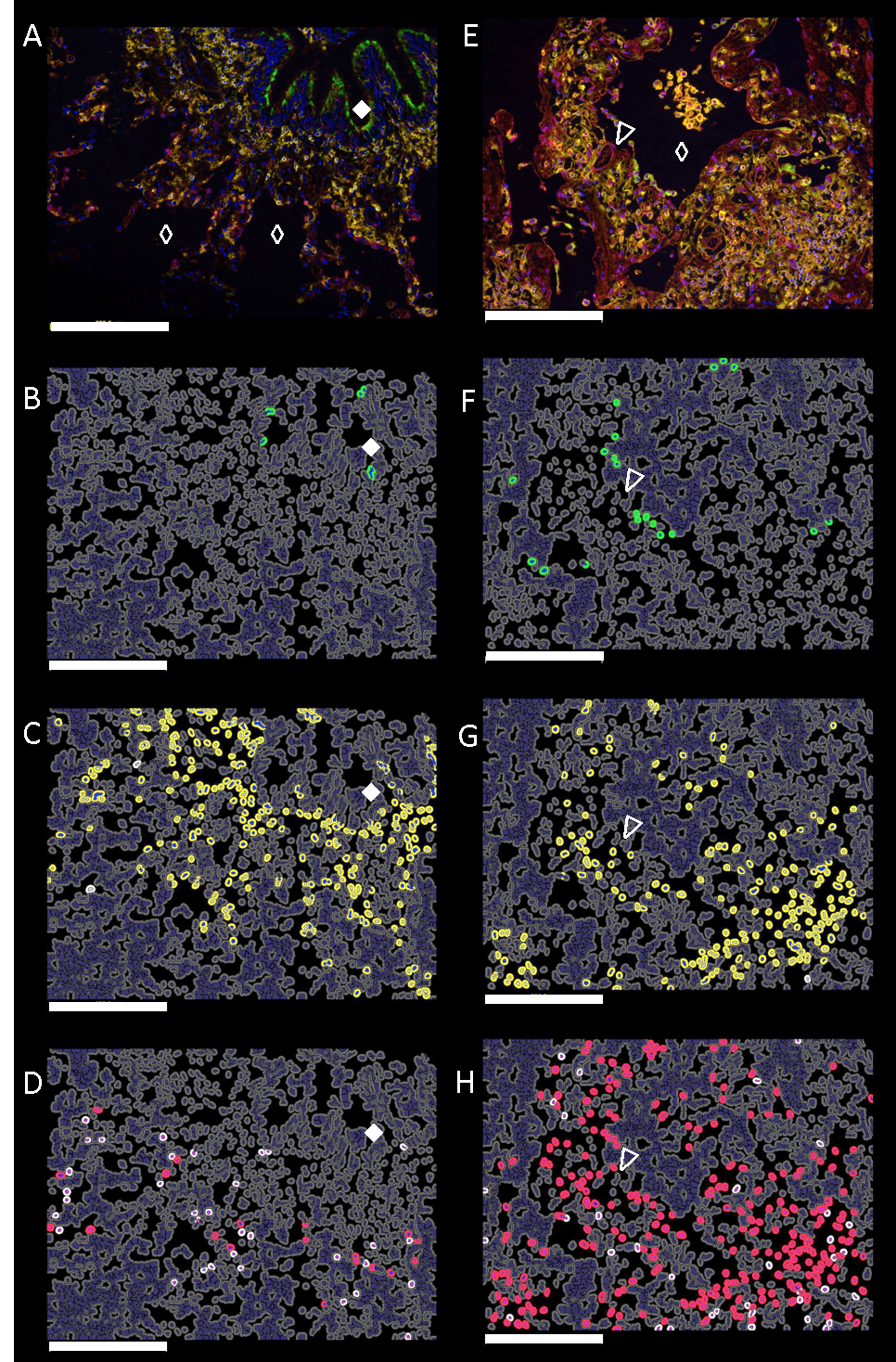

Supplement: Supplementary file 5 — Additional file 5: Figure 5. Cell phenotype quantification in CD45 panel (TIFF file). Representative analysis performed on a single 20x image of tissue from (A-D) a non-diseased control (NDC) donor and (E-F) a patient with idiopathic pulmonary fibrosis (IPF). Cells are automatically identified based on expression of Hoechst (blue) and morphological characteristics such as nuclear perimeter and roundness. Thresholds for each of the phenotype markers were set manually and positive cells were automatically identified. (A) Small airway section showing normal alveolar space (◊) and airway lumen (♦). (B) CXCR4+ cells (green) are only seen in the epithelium and (C) there is mild inflammation consisting of CD45+ cells (yellow) in subepithelial interstitial tissue. (D) CXCL12+ cells (red) are found in the alveolar walls but not in the airway. (E) In IPF tissue where alveolar space still remains, microscopic capillaries (∆) can be seen. (F) Few CXCR4+ cells can be observed within thickened alveolar septa and near capillaries. (G) Mild inflammation is also a feature in IPF as CD45+ cells are seen throughout interstitial tissue. (H) CXCL12+ cells outnumber CXCR4+ cells in IPF. Scale bar 200 μm. [file 12931_2020_1467_MOESM5_ESM.tif]

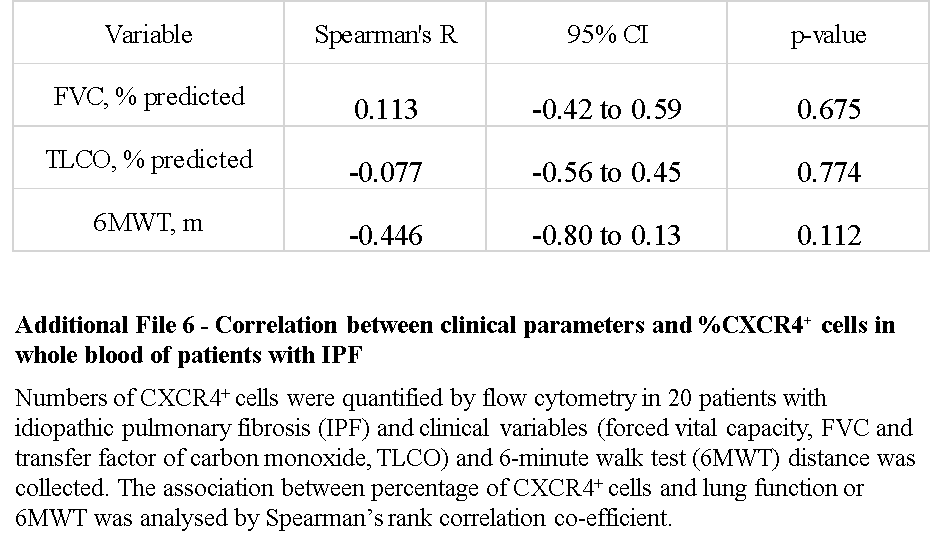

Supplement: Supplementary file 6 — Additional file 6: Figure 6. Correlation between clinical parameters and %CXCR4+ cells in whole blood of patients with IPF (PNG file). Numbers of CXCR4+ cells were quantified by flow cytometry in 20 patients with idiopathic pulmonary fibrosis (IPF) and clinical variables (forced vital capacity, FVC and transfer factor of carbon monoxide, TLCO) and 6-min walk test (6MWT) distance was collected. The association between percentage of CXCR4+ cells and lung function or 6MWT was analysed by Spearman’s rank correlation co-efficient. [file 12931_2020_1467_MOESM6_ESM.png]

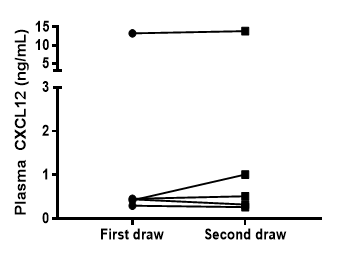

Supplement: Supplementary file 7 — Additional file 7: Figure 7. Plasma CXCL12 stable over time (PNG file). In 5 patients with idiopathic pulmonary fibrosis who had repeated bloods drawn over several months, there was no change in plasma CXCL12 level. Data are presented as mean and standard deviation. [file 12931_2020_1467_MOESM7_ESM.png]
